# Neuroendocrine carcinoma of the seminal vesicles presenting with Lambert Eaton syndrome: a case report

**DOI:** 10.1186/1752-1947-4-320

**Published:** 2010-10-12

**Authors:** Benedikt Kreiner, Stefan Denzinger, Roman Ganzer, Hans-Martin Fritsche, Maximilian Burger, Wolf F Wieland, Wolfgang Otto

**Affiliations:** 1 Department of Urology, University of Regensburg, Germany

## Abstract

**Introduction:**

Primary tumors of seminal vesicles are rare and only a few cases have been reported. Diagnosis is difficult due to the absence of early clinical signs. Prognosis is generally poor.

**Case presentation:**

We present the case of a 70-year-old Caucasian man with a seminal vesicle mass and concomitant lymph node metastasis detected by computed tomography and body positron emission tomography/low-dose computed tomography scan carried out for evaluation of Lambert Eaton syndrome. Transrectal ultrasound-guided biopsy showed a poorly differented neuroendocrine carcinoma with an immunhistochemical profile similar to small cell lung cancer. Following chemotherapy the disease was stable and active surveillance was initiated.

**Conclusions:**

Lambert Eaton syndrome may be the initial symptom of a seminal vesicle mass. Diagnosis needs to be obtained by transrectal biopsy and chemotherapy may delay progression of the tumor.

## Introduction

Primary tumors of seminal vesicles are extremely rare. A total of 51 documented cases of seminal vesicle carcinoma in men between the ages of 19 and 90 years old have been reported in the literature [1]. The first case was presented by Lyons in 1925 [2]. Epithelial and mesenchymal tumors have been described most often, while fibromas, myomas and sarcomas are found even less often [3]. Of all seminal vesicle tumors adenocarcinoma is the most prevalent [1]. Primary seminal vesicle tumors have to be clearly distinguished from a neoplasm infiltrating from outside, e.g. prostate, rectal or bladder cancer. Tumors of seminal vesicle origin must be negative for prostate specific antigen (PSA), prostatic acid phosphatase (PAP) and preferably carcinoembryonic antigen to be distinguished from prostatic and colorectal carcinomas [1,4,5].

Seminal vesicle neoplasms are often difficult to diagnose, generally presenting as a retrovesical mass that can be detected by digital rectal examination and transrectal ultrasound. However, in roughly 30% of patients no abnormalities on digital rectal examination are detected due to concomitant benign prostatic hyperplasia obscuring the seminal vesicle tumor, or to location of the tumor in the seminal vesicles are found [1]. In addition, computed tomography (CT) and magnetic resonance imaging (MRI) improve the assessment of seminal vesicle pathology.

Prognosis of patients with a seminal vesicle tumor is generally poor. Early diagnosis may result in long-term palliation or even cure. To date, no histopathological prognostic factor could be identified and the estimate of prognosis is challenging. Smith *et al. *state that seminal vesicle carcinoma can run a rapidly fatal course or possess potential for cure [6]. Surgical procedures range from local excision of a seminal vesicle to pelvic exenteration. As an adjuvant treatment, radiotherapy, chemotherapy and hormonal manipulation are debated.

## Case presentation

A 70-year-old Caucasian man was transferred to our department from the Department of Neurology where he was an inpatient due to Lambert Eaton myasthenic syndrome (LEMS).

LEMS is an autoimmune disease of the neuromuscular junction, characterized by proximal muscle weakness, areflexia and autonomic dysfunction. Antibodies are found, directed against P/Q-type voltage gated calcium channels in the pre-synaptic nerve terminal [7]. LEMS is rare with a prevalence of about 1 per 100,000, equally common in men and women. The onset is generally over the age of 50, although it can affect children [8].

Lambert, Eaton, and Rooke in 1956 described a myasthenic syndrome in association with lung cancer [9]. Small cell lung carcinoma (SCLC) is found in over half of patients [10]. There are also reports of an association with sarcoidosis and other autoimmune disorders including vitiligo. There is an association with HLA-B8 and DR3 [8]. Due to the paraneoplastic etiology of LEMS, CT is recommended at diagnosis.

In our case muscle weakness, an atactic gait, diplopic images, dysphagia and ptosis were present. In retrospective view of our patient, the first symptoms of LEMS were already present two and a half years earlier. At that time our patient did not undergo medical examination and no further diagnostic workup was initiated.

After presenting at the Department of Neurology other possible causes of LEMS were ruled out; internal investigation was performed with findings of a hypercholesterolemia. The medical history of our patient contained a transurethral resection of the prostate gland three years prior to treatment in our institution with unsuspicious histological findings showing a chronic prostatitis and benign hyperplasia. Additionally, our patient was a smoker of 60 pack-years. There was no evidence of autoimmune disease in the medical history. Currently, no lower urinary tract symptoms and no loss of weight or loss of appetite were described. The clinical and ultrasound examinations were normal.

Subsequent radiologic evaluation by CT scan of the cranium, thorax and abdomen did not show any sign of malignant disease.

One year previously our patient was first sent to our department for urogenital tumor search. No suspicious findings were detected on digital rectal examination, like the whole clinical and ultrasound examination of the urogenital tract. PSA level was 0.8 ng/mL. To complete the diagnosis a 10 core biopsy of the prostate gland was performed that showed chronic prostatitis and benign hyperplasia.

**Figure 1:**
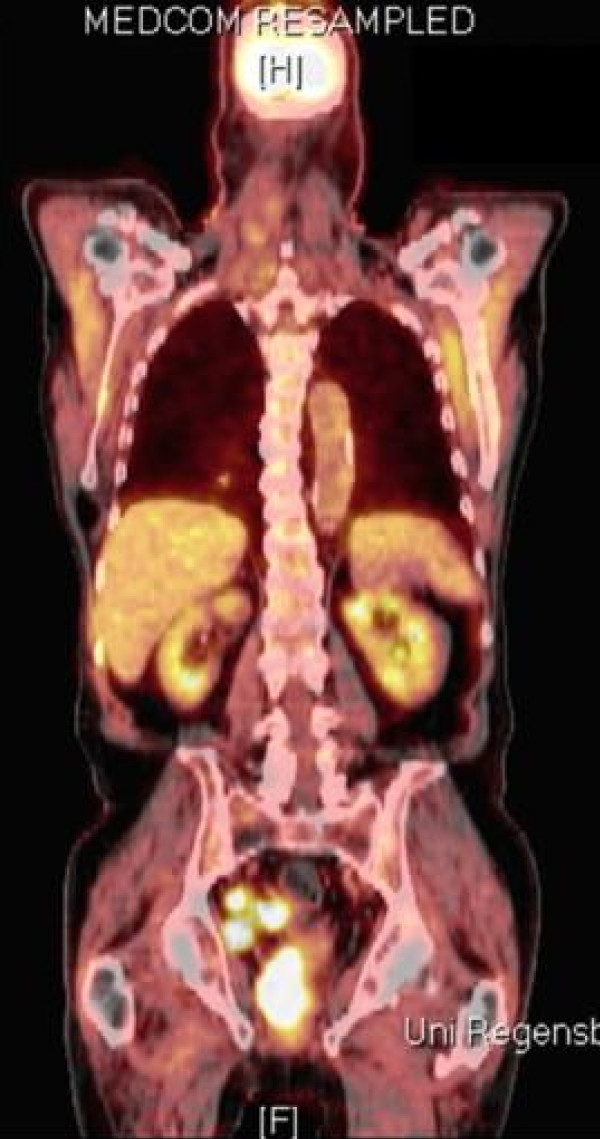
**PET-CT scan, January 2008: Strong accumulation in projection to the right seminal vesicle and the right parailiacal lymph nodes**.

Whole body positron emission tomography (PET)/low-dose CT scan (Figure 1) revealed a 2.3 × 1.9 cm tumor of the right seminal vesicle presenting as an irregular circumscribed mass. Furthermore, one lymph node lateral to the tumor and numerous para-aortal and right para-iliacal lymph nodes suspicious for metastases were found. Additionally, a circumscribed mass of 2.5 × 2.3 cm was detected in close contact to the psoas major muscle. Cranium and thorax were clear of neoplasms on CT and PET-CT.

Following the imaging results another transrectal ultrasound-guided 10 core biopsy of the suspicious right seminal vesicle and the right prostate gland was performed.The histological findings of the biopsy of the right seminal vesicle showed a poorly differentiated neuroendocrine carcinoma with immunohistochemistry similar to SCLC. The immunohistochemistry was negative for PSA, positive for chromogranin A, synaptophysin and CD99. Transcription termination factor TTF-1 was positive in about 60% of the tumor cells. A biopsy of the right prostate gland showed chronic prostatitis and a benign hyperplasia.

Having discussed all treatment options, our patient underwent six cycles of chemotherapy with carboplatin (520 mg) and etoposide (210 mg) at the Department of Hemato-oncology. Additionally pyridostigmine bromide 60 mg twice daily and 90 mg during the night for the myasthenic symptoms were initiated. With this medication the pronounced weakness that he initially presented with declined.

**Figure 2:**
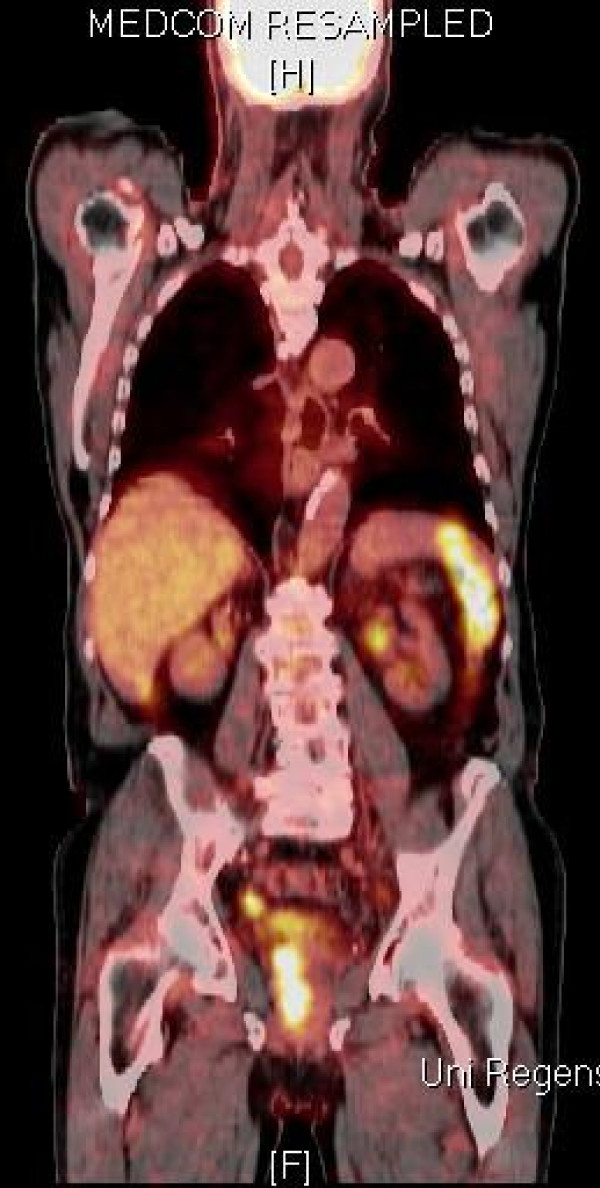
**PET-CT scan, August 2008: declining accumulation in projection to the right seminal vesicle and the right parailiacal lymph nodes**.

Following six cycles of chemotherapy, our patient presented again to our department for re-evaluation. Staging was completed by a PET-CT scan (Figure 2), a CT scan of the cranium, thorax and abdomen, and a transrectal ultrasound.

The suspicious, enlarged para-aortal and para-iliacal lymph nodes were stable. There was no evidence of new metastases. A PET-CT scan showed a declining accumulation in projection to the described para-aortal and para-iliacal lymph nodes on both sides. Also a declining accumulation in projection to the seminal vesicle was described. The transrectal ultrasound of the prostate and the seminal vesicles detected a hypoechoic 2.0 × 1.7 cm tumor in the right seminal vesicle. The biological marker neuron-specific enolase declined from 72 μg/L to 22 μg/L.

In cooperation with the Department of Hemato-oncology, we decided a strategy of observation. Our patient will re-present for re-evaluation for a two month follow-up. In case of impairment of the clinical symptoms, monotherapy with etoposide would be initiated.

## Discussion

Primary tumors of seminal vesicles are rare. In the present case a poorly differented neuroendocrine carcinoma, a very rare entity, was confirmed by histopathology. While this entity is known for sporadic primary tumors of the prostate gland, cases of seminal vesicle involvement are reported. Our patient presented with symptoms of LEMS therefore a complete neurological, internal and radiological investigation was carried out. Autoimmune diseases could be excluded along with other neoplasms. CT and PET-CT scan showed a tumor of the right seminal vesicle as an irregular circumscribed mass. Numerous lymph nodes suspicious for metastasis were detected. Taking imaging results of the CT scans and the PET-CT scans with the results of a mass in the right seminal vesicle and ultrasound and subsequent biopsy findings into consideration, a primary neuroendocrine carcinoma of the seminal vesicle was diagnosed.

The aim of treatment of primary seminal vesicles is curative radical surgery prior to any infiltration of neighboring organs or even a metastatic disease. However, hormonal manipulation and radiotherapy seem to be effective as adjuvant treatment modalities [1]. Due to the fact of histological findings of a poorly differented neuroendocrine carcinoma with immunohistochemistry similar to SCLC and the case of an advanced diseases with evidence of lymph node metastasis we decided to perform chemotherapy with carboplatin and etoposide.

While neuroendocrine carcinomas of the lung have been described in association with LEMS, the present case is the first description of this entity deriving from seminal vesicles. Therapy of poorly differented neuroendocrine carcinoma is equivalent to therapy of SCLC. Chemotherapy with carboplatin and etoposide is an accepted treatment of choice for patients with advanced SCLC [10]. Alternatively, a combination of gemcitabine and carboplatin is possible. A randomised trial showed gemcitabine/carboplatin as effective as carboplatin/etoposide in terms of overall survival and progression-free survival and has a toxicity profile more acceptable to patients [10].

## Conclusions

Seminal vesicle tumors are rare. To the best of our knowledge, we present the first case of a patient with poorly differentiated neuroendocrine carcinoma of the seminal vesicle diagnosed due to his presentation with LEMS. The prognosis of a neuroendocrine carcinoma with lymph node metastases is poor. In our case chemotherapy with carboplatin and etoposide led to stable disease, which was followed up by active surveillance.

## Consent

Written informed consent was obtained from the patient for publication of this case report and any accompanying images. A copy of the written consent is available for review by the Editor-in-Chief of this journal.

## Competing interests

The authors declare that they have no competing interests.

## Authors' contributions

BK and WO drafted the case report, SD, RG, HMF and MB helped to draft the report. WFW supervised drafting the report.
